# Social Infrastructure and the Alleviation of Loneliness in Europe

**DOI:** 10.1007/s11577-023-00883-6

**Published:** 2023-05-04

**Authors:** Christopher S. Swader, Andreea-Valentina Moraru

**Affiliations:** 1 Department of Sociology, Lund University, Sandgatan 11, Hus G, 22100 Lund, Sweden; 2 Ludwig Maximilian University of Munich, Institute for European Ethnology and Cultural Analysis, Oettingenstr. 67, 80538 Munich, Germany

**Keywords:** Public welfare, Internet access, Volunteering, Values, Fuzzy-set QCA, Gemeinwohl, Internetzugang, Freiwilligenarbeit, Werte, Fuzzy-set-QCA

## Abstract

In Europe, individualist societies, in which people more highly value independence, have fewer people who are lonely. Yet these societies also have more people who live alone, a strong determinant of loneliness. Evidence suggests that some unrecognized societal-level resources or characteristics can explain this.

We uncover multiple pathways toward a lower degree of loneliness among European societies using an ideal method for this purpose, fuzzy-set qualitative comparative analysis. Using data from the 2014 wave of the European Social Survey and other sources, we analyzed loneliness outcomes among 26 European societies. Our findings suggest two necessary conditions for a low degree of loneliness: high internet access and high association participation. Further, three pathways are sufficient for achieving less loneliness at the societal level. Most societies that have less loneliness follow both the welfare support and cultural support pathways. The third path, commercial provision, is mutually exclusive with welfare support because the former requires a weak welfare state.

The surest policy for building societies that have lower rates of loneliness includes the expansion of internet accessibility, the fostering of civil society through association participation and volunteering, and a welfare state that protects potentially vulnerable populations while funding opportunities for social interaction. This article further contributes methodologically by demonstrating “configurational robustness testing,” a more comprehensive means to implement current best practices for fuzzy-set qualitative comparative analysis robustness testing.

## Abstract (de)

Individualistische Gesellschaften in Europa, in denen die individuelle Unabhängigkeit höher geschätzt wird als in anderen Gesellschaften, haben einen geringeren Anteil an Menschen, die einsam sind. Allerdings gibt es in diesen Gesellschaften auch mehr Alleinlebende, eine Lebensform, die Einsamkeit fördert. Es gibt Hinweise darauf, dass bislang unbeachtete gesamtgesellschaftliche Ressourcen oder Merkmale dieses Paradox erklären können.

Wir nutzen eine *Fuzzy-set qualitative vergleichende Analyse*, um Pfade aufzudecken, die das Ausmaß an Einsamkeit in europäischen Gesellschaften reduzieren. Wir verwenden Daten aus der Welle 2014 des European Social Survey und weiteren Quellen, um die Folgen von Einsamkeit in 26 europäischen Gesellschaften zu analysieren. Unsere Ergebnisse deuten auf zwei notwendige Bedingungen für ein geringes Maß an Einsamkeit hin: ein weitgehend flächendeckender Internetzugang und ein hohes Engagement in Vereinen. Darüber hinaus identifizieren wir drei hinreichende Pfade, die auf gesellschaftlicher Ebene mit einem geringeren Maß an Einsamkeit verbunden sind. Die meisten Gesellschaften, die ein geringeres Ausmaß an Einsamkeit aufweisen, folgen zwei Pfaden, indem sie eine vergleichsweise hohe wohlfahrtsstaatliche als auch kulturelle Unterstützung anbieten. Der dritte Pfad, eine gut ausgebaute kommerzielle Infrastruktur, setzt einen schwachen Wohlfahrtsstaat voraus und schließt damit ein hohes Maß an Wohlfahrtsunterstützung aus.

Die sichersten politischen Maßnahmen, um das Ausmaß an Einsamkeit in einem Land zu verringern, umfassen die Ausweitung des Internetzugangs, die Förderung der Zivilgesellschaft durch Vereinsbeteiligung und Freiwilligenarbeit sowie wohlfahrtsstaatliche Maßnahmen, die potenziell gefährdete Bevölkerungsgruppen schützen und gleichzeitig Möglichkeiten zur sozialen Interaktion finanzieren. Darüber hinaus leistet dieser Artikel einen methodischen Beitrag, indem er die Anwendung „konfigurative Robustheitstests“ demonstriert, ein umfassenderes Mittel zur Implementierung aktueller Best Practices für Robustheitstests mit Fuzzy-set qualitativer vergleichender Analyse.

## Introduction

Loneliness had become a matter of public concern in the mainstream media and policy circles in Europe by 2016, when a special commission in the United Kingdom investigated the issue, leading to the establishment of a “Minister of Loneliness” by 2018. The topic saw a further boost in public salience with the COVID-19 pandemic because various countermeasures led to a breach in daily taken-for-granted social interactions.

Yet loneliness as a topic of scientific research has been somewhat neglected by sociologists over the past decades, despite empirical work especially by scholars based in the Netherlands. Scientists’ understanding of the macro level of loneliness, including its cultural dimensions and possible policy responses, is still lacking.

This article addresses the question, “Why do individualist societies have the lowest rates of loneliness in Europe?” It thus contributes toward building a theory that connects loneliness with modern culture and societal composition. Such knowledge can support a more scientifically grounded loneliness policy.

Loneliness is central to understanding the wider issue of social integration. Social integration can be defined as the overarching state of society that results from a “*certain quality *of the relations between all individuals” (emphasis added, Grunow et al., this issue). This definition has macro- and micro-level components, and loneliness is central to both. Our work addresses both the core of this definition as well as its determinants.

Defined as *the subjective perception that one’s personal relationships are lacking*, loneliness reflects the “relationship quality” heart of the micro-level dimension of social integration. Loneliness can take diverse forms, such as the perceived lack of intimacy gained from close interpersonal relationships (“emotional loneliness”) or one’s own feeling of a lack of integration with the society at large (“social loneliness”) (Weiss 1974). From the starting point of social integration defined above, loneliness is thus a negative, subjective, micro-level assessment of a “certain quality” of relations. If we instead conceptualize the “relations” in this definition as going beyond subjective feelings, loneliness is crucial because features of people’s actual social ties are among its main determinants. Loneliness thus represents people’s perceived poor quality of relationships, and its key determinants are those relationships themselves.

Besides this individual-level centrality of loneliness for social integration, loneliness also relates to the concept’s macro level, the corresponding “overarching state of society.” In this respect, this article argues both that high societal levels of loneliness constitute a low level of social integration and that the solution to this problem lies* at the macro level* because loneliness is impacted by the social, political, and cultural environments in which it is situated.

Yet a puzzle stands in the way. Loneliness has well-established individual-level determinants, such as living alone, but the cultural, societal-level determinants are poorly understood. Most research concurs that individualist societies—in which people, on average, more highly value their personal freedom and independence from family—have much lower levels of loneliness than collectivist societies, despite having more people who live alone (Lykes and Kemmelmeier 2014; Swader 2019; Heu et al. 2021; for a counterexample, see Taniguchi and Kaufman 2021). It is suggested that unknown *societal-level characteristics *of these societies correspond to them being “less lonely” (Swader 2019). A complication is that such societies tend to share many features that could feasibly explain this: They tend to have more wealth, more developed welfare states, more enhanced commercial sectors, different population structures, more tolerance toward people with diverse lifestyles, more trust in strangers, and different patterns of social practices (e.g., different rates of civil society involvement), and it is possible that they also have greater stigma related to expressing feelings of loneliness. Disentangling these effects within a relatively small sample of countries requires a specialized analysis, one that has not yet been conducted.

Our aim is therefore to clarify why particular societies in Europe are less lonely by laying out the different constellations of factors that are involved in mitigating loneliness. We ask, “How do different forms of social infrastructure combine with one another and with micro-level social composition in order to impact loneliness outcomes in Europe?”

From a policy perspective, it is less relevant for a given society to know which factors at the macro level may be impacting loneliness *on average*. Such information, provided by standard regression approaches, hides that there may very well be different combinations of conditions, *different pathways*, that equate with a low degree of loneliness. In other words, we need a more context-sensitive method that can contribute toward building theory for a more nuanced loneliness policy. We therefore conducted a country-level fuzzy-set qualitative comparative analysis (fsQCA) analysis of 26 societies sampled in the European Social Survey (ESS). The data consist of aggregated ESS data and a range of other country-level data. In this way, this article contributes to theory by revealing the main pathways by which societal individualism equates with reduced loneliness in Europe.

## Theory and Previous Research

### Loneliness Research

Loneliness is a fundamental sociological concern because it involves the relational well-being of human beings, has been experienced by almost everyone, and is heavily impacted by social behavior and characteristics. However, the discipline has generally been slow to recognize loneliness as a social problem (Yang 2019). In addition, loneliness is important because of its links to a host of health problems, including depression and early death, and it has health risks greater than those associated with obesity (Holt-Lunstad et al. 2015). Loneliness has also been linked to other important social issues, such as political disengagement (Langenkamp 2021). While the COVID-19 pandemic likely enhanced both social isolation and loneliness (Bierman and Schieman 2020) as well as the public salience and wider scientific awareness of these issues, it is worth noting that loneliness and social isolation were prevalent and discussed as “an epidemic” long before COVID-19 restricted people’s access to social places (Hwang et al. 2020).

*Loneliness *is defined as the subjective feeling that one’s social relationships do not meet one’s ideals; in other words, it is* the perception of social isolation *(Weiss 1974, pp. 18–19, 34; see also Perlman and Peplau 1982). In contrast, social isolation itself is the objective state of being alone in some way (“aloneness”), for example living alone, rarely meeting with others, or having a lack of high-quality intimate (in terms of emotional exchange) relationships. In a nutshell, loneliness is *feeling* alone, while social isolation is *being* alone in some way.

By now, there is a decent understanding of how loneliness correlates with basic personal characteristics, such as age, gender, and social class. In terms of age, there appears to be a U-shaped relationship, with the very old and the very young being the most lonely, and middle-aged adults being the least lonely (Luhmann and Hawkley 2016). Regarding gender, a meta-analysis has confirmed that men are lonelier, but the substantive difference is small (Maes et al. 2016). We also know that loneliness is more prevalent among poor people, disadvantaged social classes (Rubinstein et al. 1979; Madsen et al. 2019), and other vulnerable groups, such as those who experience discrimination or unemployment (Creed and Reynolds 2001). In most cases, the above personal characteristics are not analyzed or theorized in connection to social settings or cultural dimensions in a way that highlights the macro dimension of loneliness.

### Loneliness and Culture

Over the past 20 years, research has begun to incorporate cultural dimensions and other macro-level societal features into the study of loneliness. There are many studies that report on cultural–geographic differences in loneliness, which show, for instance, that loneliness is more prevalent in Eastern and Southern Europe compared with Northern and Western Europe (Lykes and Kemmelmeier 2014; Swader 2019).

The reasons for such differences are unclear despite efforts to explain them. A recent article (Rapolienė and Aartsen 2021) works to explain high loneliness through lack of trust, which leads to reduced social engagement with others. It argues that, within totalitarian societies in Eastern Europe, people were exposed to various forms of violence during childhood, which weakened their trust in others and thus led to increased loneliness. However, this theory cannot explain the high loneliness rates found in Southern Europe nor those experienced by young people in Eastern Europe who were born after the collapse of socialist regimes.

The most promising cultural research direction has been *individualism–collectivism* (hereafter “individualism”). Individualism as a cultural pattern implies a focus on the self, independence, and one’s own goals instead of interdependence and collective goals (Hofstede 2001; Triandis 2002), and it is spurred by economic development and therefore most pronounced in rich societies (Inglehart 1989; Welzel 2013). At the macro level, under investigation in this article, *societal *individualism refers to a society in which there is an average preference for these values.

### The Puzzle: The Unexplained Societal-Level Individualism Effect

The central finding to be explained is that individualist societies—in which large portions of the population also happen to live alone—have *lower *rates of loneliness (Lykes and Kemmelmeier 2014; Swader 2019). Because living alone is a major determinant of *increased *loneliness, these two facts result in the “cultural paradox of loneliness” (Heu et al. 2021). A recent analysis using a limited sample from beyond Europe has questioned whether this pattern holds outside of Europe (Taniguchi and Kaufman 2021).

This paradox has been deepened by research findings that have challenged the most obvious solution, an individual-level explanation. Perhaps people who are individualists *value social ties less* and are for this reason less likely to feel lonely when those ties are weak or missing. Could the society-level findings showing less loneliness in individualist societies be an aggregate outcome of this? This appears to not be the case, because one’s personal degree of individualism cannot cancel out the effects of social isolation leading to loneliness. Moreover, a high degree of personal individualism may even be linked to *enhanced *loneliness (Swader 2019; see also Lykes and Kemmelmeier 2014; Heu et al. 2019), in contrast to *country-level *individualism, which appears to be strongly tied to diminished loneliness. Individualist societies are therefore less lonely due to some unknown factors *at the societal level*.

A recent contribution suggested that within collectivist societies, which have more restrictive norms, both the perception of isolation and emotional isolation are more prevalent, while individualist societies’ populations are more prone to physical isolation (Heu et al. 2021). Despite the value in such a norm-rich explanation, there are reasons to expand it.^1^ We therefore combine two features at the cultural level suggested by Heu et al. (2021), *a system of norms *and the *aggregation of various forms of isolation*, with a variety of types of *social infrastructure*. In other words, we suggest that individualist societies also contribute a set of *social resources* toward reducing loneliness (see Fokkema et al. 2012; Swader 2019).

### Solving the Puzzle with Social Infrastructure?

Another clue leads us to think that a social resource approach is needed. As mentioned, loneliness has been long recognized as more prevalent among people who are poor (Weiss 1974, p. 26; Rubinstein et al. 1979, p. 61), but there has been no established explanation. We suppose that individualist societies may contribute unknown resources toward reducing loneliness but that these may be unequally distributed, evaluated, and accessed according to socioeconomic divisions, thus leaving the poor more at risk. Scientific literature already documents the extensive inequality and exclusion entailed by the social infrastructure of cities, especially in relation to consumption opportunities (Gieryn 2000). Work on segregation within cities has identified that low-income and minority districts of cities often have severe shortages of food sources and educational opportunities; they are “food deserts” (Vaughan et al. 2017) and “education deserts” (Hillman 2016). We propose that there may also be park, cafe, and library deserts that limit the access of vulnerable groups to potential loneliness management resources.

A similar logic may be applied at the macro-societal level. Because individualist societies are wealthier than collectivist ones, rich societies may provide specific forms of social infrastructure that help individuals to manage loneliness. As they are relevant forms of social infrastructure, we will argue for the impacts of commercial and public infrastructure, cultural preferences, the role of associations, and technological development.

Not referring merely to the physical infrastructure of a place, we define social infrastructure as the social resources embedded in the underlying organizational, cultural, and constructed features of a society (Klinenberg 2018). Social infrastructure is distinct from social capital in that it allows social capital to develop and thrive (Klinenberg 2018, p. 5). For example, scholars have shown that one form of social infrastructure, social spaces, can foster social cohesion. Such places have the potential to be “communicative” (Jeffres 2010) “places of encounters” (Koutrolikou 2013; Oldenburg 1999) that fuel social life. Yet such work has underappreciated that such social infrastructure can also be instrumental for people who are struggling with loneliness. Recent work in this direction has linked locally built environments to loneliness and social isolation (Reed and Bohr 2021), but links to how social infrastructure aggregates at the macro level are missing.

### Explaining Macro-Level Loneliness Alleviation

Individualist societies tend to be wealthier, which implies more social infrastructural resources that may mitigate loneliness. However, many of these resources are found in combination, so our method works to understand the specific combinations of factors that are the most crucial.

Our approach expands the societal level of a model put forward by de Jong Gierveld and Tesch-Römer (2012) that aims to explain the interplay between social integration and loneliness. They argue for the combination of “societal wealth and welfare, demographic composition and cultural norms and values” as crucial societal-level features relevant for loneliness. We include each of these but expand the “societal wealth and welfare” dimension to include five types of social infrastructure: commercial social infrastructure (in particular, size of the restaurant and cafe sector), public social infrastructure (the welfare state), technological development (in particular, internet accessibility), civil society (people’s involvement in associations), and cultural preferences (e.g., an orientation toward the generalized other).^2^

We therefore investigated six conditions: the five social-infrastructural conditions above as well as a social compositional effect from the strength of personal relationships (see Online Appendix 1.8, Table 10, for their links to individualism).

#### Commercial Social Infrastructure

Individualist societies may possess more developed commercial social infrastructure that is dedicated to the *profitable* management of loneliness. Commercial exchange in general has adapted itself to be “hospitable” (Bell 2007), which allows it to function better as a social resource. Hospitality sells, and the field of marketing has identified “the lonely consumer” to better target products toward this group (Wang et al. 2012). “Everyday care work” (Warner et al. 2012) and important forms of social integration happen in commercialized “social spaces” while their owners profit from the exchange.

Marketplaces generally can be viewed as sites of social activity (Watson 2009), and of course, economic exchange is constituted by such activity. Shopping malls (Abaza 2001), for example, are open to the public, but financial transactions are needed to fully access their most social spaces, such as cafes, restaurants, and stores. Cafes (Warner et al. 2012; Ferreira et al. 2021; Broadway and Engelhardt 2021) and restaurants (Lugosi 2016) are among the clearest examples of places where patrons can surround themselves with others, even if alone, at the cost of what they will consume.

Commercial establishments sell forms of social immersion directly and indirectly. People who acquire social interaction through their commercial activity may feel less lonely as a result.

#### Public Social Infrastructure

Individualist societies tend to have highly developed welfare states, which may provide public social infrastructure that explains their lower rates of loneliness. One category of this is state *protective support* for individuals through, for example, unemployment support, pension support, parental and child support, and similar measures (Ackermann et al., this issue). The cross-country research of Visser et al. (2018) into the “crowding-in” hypothesis suggests that broad welfare policies foster better social interaction (in both quality and quantity) through freeing up individual resources and encouraging a more empathetic culture (Ackermann et al., this issue). In a similar vein, we suppose that such support would reduce loneliness by allowing more isolated groups—who might lack support via their personal social networks—to be both financially and cognitively supported by the wider society through their relation to the state. In addition, welfare state protection may also increase the self-reliance (i.e., resilience to loneliness) of the population (Machielse 2015). Evidence for these impacts is that Nordic welfare regimes, through their high social expenditures, have been found to have a “socially enabling” impact in relation to loneliness, which means that loneliness outcomes are less affected by individual resources and familial ties (Nyqvist et al. 2019).

States also provide direct *social capital support* activities, for instance through their funding of social places and social activities such as parks, libraries, museums, sports, and cultural and recreational activities. Such activities and places have long been recognized for their importance to wider social life (Putnam 2001), but the roles of states in funding them, for example to support disadvantaged communities (Skinner et al. 2008) or to build social capital at large (Daly 2005), have not been fully investigated in relation to loneliness alleviation.

In short, we suppose that protective support provided by welfare states relieves the feeling of loneliness, while social capital support activities impact the opportunities and (perceived) quality of social interaction among people who benefit from them. Further, we note that socially isolated people are among those who can most benefit from both of these categories of support.

#### Internet Infrastructure

Access to the internet also may alleviate loneliness because virtual worlds, including social media, social games, email, fora, and video conferencing, can be a social resource used especially by more isolated people to feel integrated. There is an empirical association between internet use and loneliness, with evidence that lonely people use the internet in order to cope (Morahan-Martin and Schumacher 2003), and this strategy may have links to depression (Demir and Kutlu 2016). However, other work suggests that the impact of internet use on loneliness depends upon whether people use it to displace offline relationships or to build new ones (Nowland et al. 2018). While internet usage has been often referred to as weakening ties between individuals, country-level evidence shows that highly developed internet infrastructure and communication technology are associated with high social cohesion (Dragolov et al. 2016). Moreover, it is also important *who* is using the internet. Longitudinal research has found that internet use is associated with somewhat less loneliness and more social contact among the elderly (Yu et al. 2021). For the above reasons, we include internet access as one of our conditions and suggest that access to the internet has the capacity to alter and build social relationships as well as impact our subjective perception of them, thereby potentially alleviating loneliness.

#### Associations

Individualist societies may be less lonely because they have a highly developed civil society that is separate from the state sector. A rich accessibility of groups of people with similar political or cultural interests or hobbies may be critical for social cohesion (Putnam 2001).

Voluntary work is an important type of associational activity. Research shows that such social leisure activities, such as “voluntary work, cultural activities, holiday, sports,” are crucial for the social integration of older adults (Toepoel 2013), and the volunteering link to reduced loneliness was confirmed in a more recent study (Lee 2021). At the same time, volunteering has been shown to increase in vulnerable populations when they are targeted with various forms of welfare support (Ackermann et al., this issue).

We presume that volunteering may alleviate loneliness both among volunteers, through their social engagement and enhanced sense of belonging, as well as among their beneficiaries, through their potential engagement with volunteers as well as through the enhanced perception that others care about them.

#### Cultural Preferences (Relational Pluralism)

There may be other cultural preferences, narrower than individualism itself, that societies possess that may mitigate loneliness. While individualism–collectivism is also a type of cultural preference, it is a wide one that overlaps with many other features. This overlap makes it difficult to pin down which specific aspects may be, as mechanisms, impacting loneliness. For this reason, we seek a more targeted cultural preference associated with individualism that can explain its effect.

Our leading candidate is “*relational pluralism*,” which—harmonious with “bridging social capital” (Putnam 2001)—indicates a wider tolerance and openness toward others who have different lifestyles (including toward those who are lonely). Such diversity-oriented cultural preferences could be linked to *more tolerance* in general for diverse lifestyles, as well as toward stigmatized conditions, such as being isolated or feeling lonely. At the same time, such cultural preferences may be linked to a greater likelihood to *directly help* others in need. People with more pluralistic cultural preferences are more likely to *establish relationships* with people who are different from themselves. Openness and trust toward diverse others can also be seen as a resource for establishing good relationships in general and thus averting loneliness (Rapolienė and Aartsen 2021).

Individualized societies are ambivalent in how their citizens value others. Their ties to others are less based on similarity and a motivation to sacrifice themselves for the needs of the wider group (Durkheim 1997; Tönnies 1988). People in such societies tend to be individualists, but this also corresponds to a wider “radius” in terms of their orientation toward others, as seen in work on trust (Delhey et al. 2011; van Hoorn 2015). Individualists are thus more focused on the *generalized other*, on strangers rather than on the intimate circle. We expect such *relational pluralism*—an openness toward diverse others—to be a resource contributing to less loneliness. We note that this concept is equivalent to a less restrictive normative culture, and thus it incorporates the macro-level normative dimension of Heu et al.’s culture-loneliness framework (Heu et al. 2021).

The value of relational pluralism should enhance loneliness by resulting in a greater quantity and diversity of social relationships as well as the perception, especially among people who are more isolated, that other people care about them.

#### The Social Composition Effect of Personal Relationships

In contrast to the above conditions, which are forms of social infrastructure embedded at the macro-cultural level within individualist societies, the “personal relationships” condition reflects a society’s *composition*. Individualist societies may be composed of populations with better (in terms of quality or quantity) relationships (despite that such societies have more people who live alone). Because these micro-level forms of social integration are linked to less loneliness, such societies may be less lonely as a result. Heu et al. (2021) similarly argue that people in individualist societies are less likely to perceive social isolation and more likely to be emotionally integrated. A lower prevalence of loneliness in such societies could be the outcome of such societies’ compositions as a result. While such an argument is theoretically compelling, a mediation analysis has shown that the country-level aggregate degree of the high quality and quantity of personal relationships explains only a modest portion (22%) of the impact of country-level individualism on loneliness (Swader 2019, p. 1327). We include this condition in our analysis because we know it is relevant but incomplete, and we do not know how it interacts with the other conditions that are part of individualist societies’ social infrastructure.

### Summary of Expectations

In line with qualitative comparative analysis (QCA) methodology, our expectations involve conditions rather than variables. We theorize each of these conditions to be sufficient, but not necessary, for a “less lonely society” because there is no substantial theory explaining a priori why any particular condition would be found in all cases where “low loneliness” is present. In contrast, as outlined above, we suspect that each of these conditions may be part of a sufficient path for less loneliness.

We list our expectations for isolated sufficient conditions but not for combinations between them. This is because little is known about how they interact in relation to their connection to less lonely societies. With a theory-building aim, the result of our analysis will outline precisely which combinations of these conditions are sufficient for the outcome, a society with a low degree of loneliness (LL).

1. A high degree of commercial social infrastructure (CI) is at least part of a sufficient combination of conditions (an “INUS” condition^3^) for a society with a low degree of loneliness because advanced economies “sell” and profit from loneliness alleviation.
2. A high degree of public social infrastructure (PI) is at least part of a sufficient combination of conditions for a society with a low degree of loneliness because welfare states provide protective support for vulnerable groups as well as social capital support for the population at large.
3. A high degree of internet accessibility (I) is at least part of a sufficient combination of conditions for a society with a low degree of loneliness because the internet is an important social resource used by people to feel integrated.
4. A high degree of participation in associations (A) is at least part of a sufficient combination of conditions for a society with a low degree of loneliness because this type of grassroots, nonstate activity is an important means for creating community, generating new social ties, and strengthening old ones.
5. A high degree of relational pluralist cultural preferences (CP) is at least part of a sufficient combination of conditions for a society with a low degree of loneliness because this indicates wider societal tolerance toward diverse others and a stronger proclivity to socialize with and to help others beyond one’s own immediate group memberships.
6. Strong personal relationships (P) are at least part of a sufficient combination of conditions for a society with a low degree of loneliness because there are strong links between various forms of social isolation and perceived loneliness. This is equivalent to arguing that less lonely societies are this way because they are composed of populations that are more socially integrated at the micro level and are thus less susceptible to loneliness.

## Methods

### Our Approach

The choice of the fsQCA method is appropriate for three reasons. First, we are interested in a more complex understanding of potential causal pathways and reject *as a starting position* that there may be some condition that is valid on average for less lonely societies. Because individualist societies share so many traits, we expect multiple pathways.

Second, our outcome of low loneliness among European societies is conceived of as a set. Previous research had revealed a particular group of societies to be “less lonely,” and we want to know why. Qualitative comparative analysis will allow us to outline the necessary and sufficient combinations of conditions that are associated with this outcome. This will allow us to contribute a more nuanced and contextualized understanding of the different ways in which individualist societies in Europe are able to “manage” loneliness. This also highlights that QCA contributes toward theory building.

Third, we have 26 countries in our data set, and QCA is well-known for its ability to handle small‑N and intermediate‑N sample sizes, which are less suited for more traditional statistical approaches (Schneider and Wagemann 2012, p. 12). Qualitative comparative analysis has been used once before in relation to loneliness (Yang 2018), but that study was an individual-level analysis, and it did not concern cultural features.

### QCA in a Nutshell

This section serves as a snapshot, for less familiar readers, of the logic of QCA. For a deeper dive into QCA, we recommend works such as those by Ragin (2008), Ragin and Rihoux (2009), Schneider and Wagemann (2012), and Dușa (2019). As a note to experts, in order to maintain accessibility for a wider audience, we do not adopt all the standard conventions of QCA terminology and presentation style within the main text. Explicit details, explanations, and Boolean expressions can be found either in footnotes or within the extensive Online Appendix.

Qualitative comparative analysis explains phenomena by drawing logical conclusions derived from the “memberships” of cases within specified conditions and outcome sets (Mill 1851; Ragin 1987). Within fsQCA, which we use, a case can rank anywhere on the continuous scale of 0 to 1, where 0 means “fully out” of a set, 0.5 means that set membership is not defined, 1 means “fully within” the given set, and 0.75 would mean “more in than out of a set” (Ragin 2008, pp. 29-33). The original variation between cases across the conditions and the outcome is maintained in this fuzzy-set variant. Membership scores are established by the researchers within a “calibration” process (Online Appendix 1). Afterward, the analysis determines the necessity and then sufficiency of combinations of conditions in relation to the outcome.^4^

Qualitative comparative analysis can be distinguished through the principles of equifinality, asymmetry, and conjunctural causation. These combine to form a methodology that embraces the validity of multiple pathways toward the same outcome (“equifinality”). Those pathways are (usually) composed of combinations of conditions (“conjunctural causation”). A condition can be insufficient/unnecessary for an outcome *on its own *but still be an important part of the solution in combination with another condition (Schneider and Wagemann 2012, pp. 78-79). “Asymmetry” adds to this the fact that combinations that are established for an outcome are valid for that outcome alone but not for its negation, for which a separate analysis is conducted.^5^

Qualitative comparative analysis differs from statistical methods in that it does not make inferences about a wider population based on a random sample. Rather, it richly describes the logical connections between outcomes and conditions that are observed within a given set of cases and thus contributes to building a theory that explains those cases. Inferences made about unmeasured cases need to be justified on theoretical grounds—similar to the “analytic” mode of generalization within qualitative social scientific research (Halkier 2011)—rather than based on the assumption that a sample directly informs about the population from which it is drawn.

### Our Cases

**Fig. 1:**
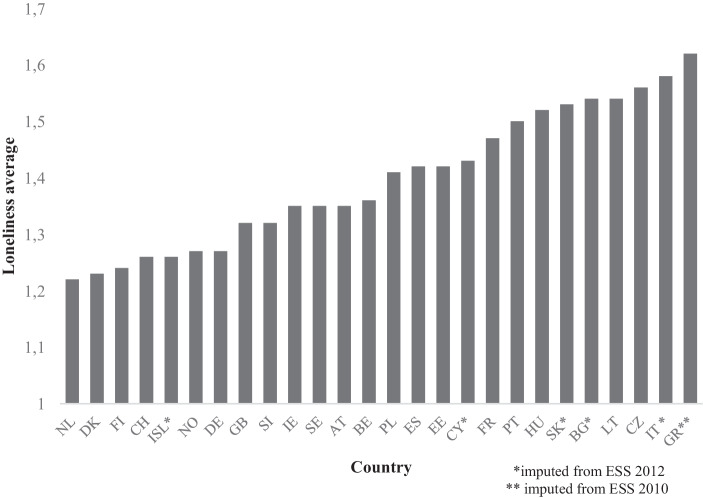
Average levels of loneliness in Europe (ESS 2014). Loneliness indicates how often the individual felt lonely in the past week (weighted average; 1 = none, 2 = some of the time, 3 = most of the time, 4 = almost all of the time). *AT* Austria, *BE* Belgium,* BG* Bulgaria, *CH* Switzerland, *CY* Cyprus, *CZ* Czechia, *DE* Germany, *DK* Denmark, *EE* Estonia, *ES* Spain, *FI* Finland, *FR* France, *GB* Great Britain, *GR* Greece, *HU* Hungary, *IE* Ireland, *ISL* Iceland, *IT* Italy, *LT* Lithuania, *NL* The Netherlands,* NO* Norway, *PL* Poland, *PT* Portugal, *SE* Sweden, *SI* Slovenia, *SK* Slovakia

The ESS has the most reliable cross-country sample that covers loneliness. Therefore, we used all available countries of the ESS for the last wave in which loneliness questions were found, in 2014, and we gained additional cases by imputing from the 2012 and 2010 waves (Online Appendix 1.1). The data set therefore consists of 26 countries, which are displayed in Fig. 1 in terms of their mean levels of loneliness.

### Operationalization

Our outcome condition, low loneliness, is calculated from the (poststratification weighted) country-level average of the ESS question *“How much of the time have you felt lonely in the past week?”* from 2010, 2012, and mainly 2014 (ESS 2010, ESS 2012, ESS 2014). Respondents could answer *“None or almost none of the time,” “Some of the time,” “Most of the time,” or “All or almost all of the time” *(Online Appendix 1.1). The set “less lonely” is defined in relation to the European cases included in the sample, in which there is a gap especially between Northern and Western Europe—which tend to have a lower degree of loneliness — and Eastern and Southern Europe. The precise fuzzy-set calibrations of this outcome and all conditions are found in Online Appendix 1.

High commercial infrastructure (CI) was operationalized as how much the citizens of a given society spend per capita on restaurants and cafes, adjusted to euros using purchasing power parity (Online Appendix 1.2). The consumption of restaurant and cafe services represents our interest in commerce’s role—through shops, cafes, and restaurants—in selling social interaction and thus the service of loneliness amelioration.

For high public infrastructure (PI), we measured this through a Boolean multiplicative index in which societies within this set have a high amount of per capita spending both on the social protection (Eurostat 2021c) of vulnerable populations and on social capital support: the promotion of culture, recreation, and sports (Eurostat 2021a, b). The indicator represents the welfare state’s dual functions of protecting the vulnerable (including the potentially lonely) and providing opportunities for people to meet one another.

The measurement for high internet accessibility (I) is a Eurostat indicator indicating the percentage of households with internet access in 2014 (Eurostat 2021d). Internet penetration indicates whether even more marginalized people have access to others through the internet.

We measured high participation in associations (A) through an indicator for volunteering. The ESS 2012 included a question about the frequency of volunteering over the past year, and we were interested in people who volunteered at least once per month (ESS 2012; Online Appendix 1.5). Research has demonstrated the connection between volunteering and reduced loneliness (Lee 2021).

High cultural preference (CP) measures peoples’ cultural values for understanding and supporting diverse others, *relational pluralism*. We have operationalized this through the weighted country averages of the well-established Schwartz (2012) indicator on universalism: *“It is important to [them] to listen to people who are different from [them]. Even when she/he disagrees with them, [she/he] still wants to understand them*” (ESS 2014). Individuals could answer on a six-point scale ranging from *“very much like me*” to *“not like me at all*.” This indicates the respondent’s orientation especially toward those who are in an outgroup, thus indicating the modern pluralism embedded within individualist values.

We operationalized strong personal relationships (P) through a two-component additive index made up of indicators for qualitative and quantitative social ties, respectively involving how many people the respondents could *“discuss intimate or personal matters*” with and how often they *“socially meet with friends, family, colleagues*.” We took these as the weighted country averages from ESS 2014, with missing values imputed from the 2012 and 2010 ESS waves.

### Analysis Steps

Following the fsQCA steps provided by Schneider and Wagemann (2012), we began with a necessity analysis and followed this with a sufficiency analysis.^6^ We followed with the enhanced standard analysis procedure (Schneider and Wagemann 2012), which is detailed further in Online Appendix 2.3. We then described the pathways toward low loneliness in Europe that we have found. Thereafter, we ran a separate analysis on the negated outcome, the lack of low loneliness. All results are discussed in depth in an integrated fashion in the discussion section.

### Configurational Robustness Testing

Fuzzy-set QCA has rigorous robustness traditions involving robustness checks across at least three dimensions. We implemented an enhanced form of robustness testing that tests *all combinations *of case robustness, theoretically valid calibration thresholds, and truth table row consistency thresholds. This *“configurational robustness testing” *resulted in 17,280 unique theoretically valid combinations of analysis choices and 67,788 individual solution models. This new procedure is consistent with the state of the art in robustness testing (Oana and Schneider 2021), except that our approach advances the field by considering only conceptually valid choices and maps out the complete range of robustness choice *combinations *instead of each of the three robustness choices in isolation (Online Appendix 3).

## Results

### The Necessity of High Internet Access and High Participation in Associations

The “necessity test” finds both high association participation and high internet access to be necessary, and substantively relevant, conditions for low loneliness (Online Appendix 2.2). These, especially high internet access, are by far the most common “necessary conditions” found in robustness testing (Online Appendix 3.4). This finding has the further implication that Slovenia, which is “less lonely,” becomes unexplained because it lacks high internet access.

### Which Conditions Are Sufficient for Societies to Be Less Lonely?

The *truth table* for this sufficiency analysis (Table 1) displays the presence or absence of conditions and whether their particular combination in each row is sufficient for low loneliness. The analysis includes an assessment of the cases in each row as well as the row’s consistency score (“Incl” in Table 1), which should be greater than 0.75 and ideally closer to 1 (Schneider and Wagemann 2012, p. 278).

**Table 1:** Truth table displaying which combinations of conditions are sufficient for low loneliness

Row	CI	I	A	CP	P	PI	OUT	n	Incl	PRI	Cases
64	1	1	1	1	1	1	1	2	0.948	0.896	CH, AT
63	1	1	1	1	1	0	1	2	0.945	0.894	ISL, GB
31	0	1	1	1	1	0	1	1	0.940	0.898	DE
32	0	1	1	1	1	1	1	4	0.938	0.906	DK, SE, BE, FI
57	1	1	1	0	0	0	1	1	0.877	0.672	IE
28	0	1	1	0	1	1	1	2	0.864	0.782	NL, NO
13	0	0	1	1	0	0	0	1	0.833	0.576	SI
*5*	*0*	*0*	*0*	*1*	*0*	*0*	*0*	*1*	*0.728*	*0.264*	*PL*
*47*	*1*	*0*	*1*	*1*	*1*	*0*	*0*	*1*	*0.717*	*0.248*	*ES*
*45*	*1*	*0*	*1*	*1*	*0*	*0*	*0*	*1*	*0.699*	*0.221*	*IT*
*26*	*0*	*1*	*1*	*0*	*0*	*1*	*0*	*1*	*0.673*	*0.237*	*FR*
*35*	*1*	*0*	*0*	*0*	*1*	*0*	*0*	*1*	*0.645*	*0.093*	*PT*
*17*	*0*	*1*	*0*	*0*	*0*	*0*	*0*	*1*	*0.535*	*0.113*	*EE*
*37*	*1*	*0*	*0*	*1*	*0*	*0*	*0*	*2*	*0.513*	*0.051*	*CY, EL*
*1*	*0*	*0*	*0*	*0*	*0*	*0*	*0*	*5*	*0.306*	*0.021*	*HU, SK, BG, LT, CZ*

The rows in italics contain cases in which the outcome of low loneliness (LL) is absent. Note that the OUT column contains different information from this: It pertains to whether a particular truth table row is a sufficient combination for the outcome. The row containing Slovenia is declared to be an insufficient combination for LL because it lacks the necessary condition of I. Also, there is no row deemed sufficient that has a case that does not possess the LL outcome

*CI* high commercial infrastructure (service sector), *I *high internet accessibility, *A* high involvement in associations (volunteering), *CP* high cultural preferences for universalism (relational pluralism), *P* strong personal relationships, *PI* high public infrastructure, *OUT* output, *Incl* inclusion score (also known as consistency), the extent to which this row’s combination of conditions is logically consistent with a “sufficient” set relationship with the outcome of LL, having a range from 0 to 1, *PRI* proportional reduction in inconsistency, indicating the extent to which a set relation is consistent for the outcome (1) compared to the outcome’s negation (0)

Cases: *AT* Austria, *BE* Belgium,* BG* Bulgaria, *CH* Switzerland, *CY* Cyprus, *CZ* Czechia, *DE* Germany, *DK* Denmark, *EE* Estonia, *ES* Spain, *FI* Finland, *FR* France, *GB* Great Britain, *GR* Greece, *HU* Hungary, *IE* Ireland, *ISL* Iceland, *IT* Italy, *LT* Lithuania, *NL* The Netherlands,* NO* Norway, *PL* Poland, *PT* Portugal, *SE* Sweden, *SI* Slovenia,* SK* Slovakia

Rows that have a 1 in the OUT column are considered valid pathways for the outcome. Thereafter, we followed the enhanced standard analysis procedure (Schneider and Wagemann 2012, Chap. 8.2) to decide how to handle rows for which no cases are present, and a “logical minimization” process simplified our results into three pathways (extensive details on the analysis can be found in Online Appendix 2.3).

**Fig. 2:**
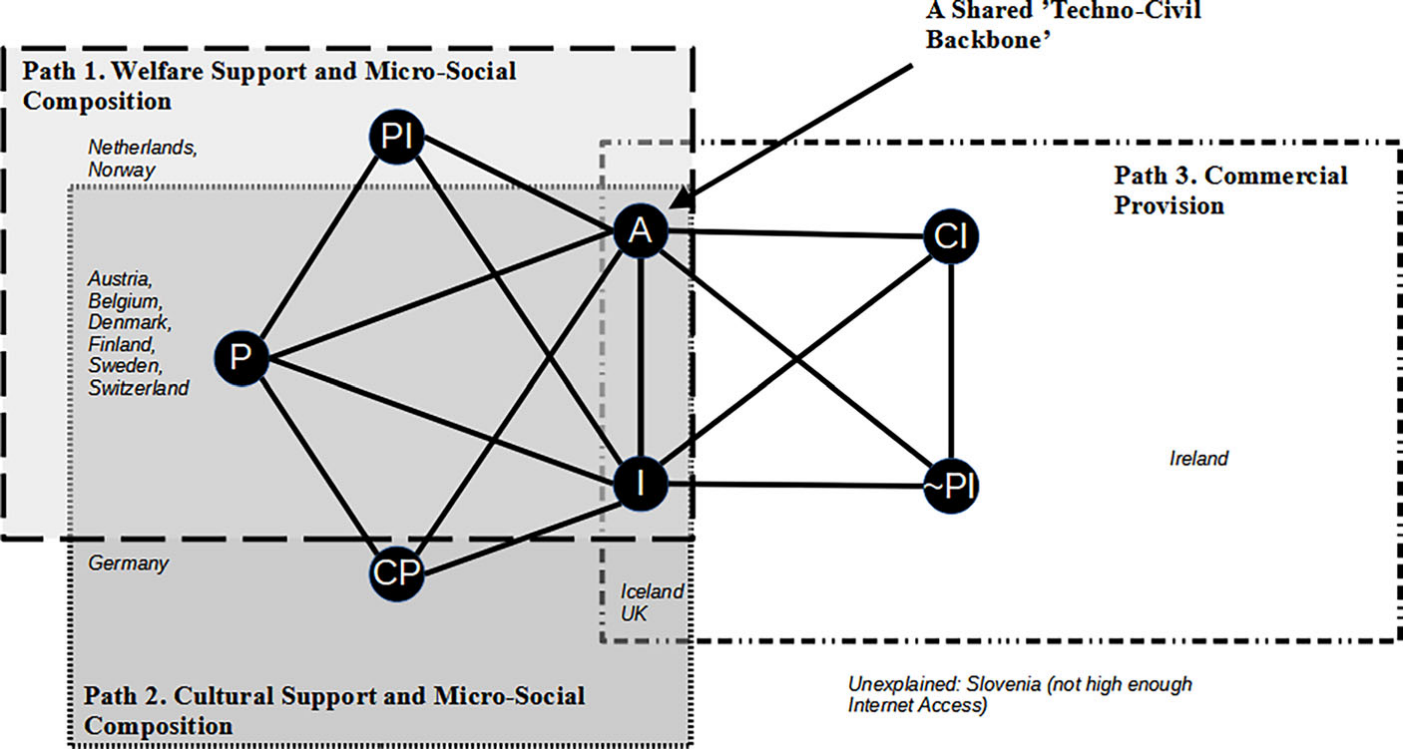
Pathways toward low loneliness: network of sufficient combinations of conditions. Solution: I*A*P*PI + I*A*CP*P + CI*I*A*~PI → LL. *I* high internet accessibility, *A* high involvement in associations (volunteering), *P* strong personal relationships, *PI* high public infrastructure, *CP* high cultural preferences for universalism (relational pluralism), *CI* high commercial infrastructure (service sector), *~* *PI* lack of high public infrastructure, *LL* low loneliness

The main outcome of our sufficiency analysis is that we have found *three sufficient pathways* (combinations of conditions) for the outcome of low loneliness (Fig. 2 and Online Appendix 2.3.3).^7^ These include the welfare support, cultural support, and commercial provision pathways. They leave only Slovenia unexplained, have no “true logical contradictions,” and are supported by robustness testing (Online Appendix 3). In other words, each society in Europe that is less lonely, except for Slovenia, has one of these three combinations of conditions.

Within QCA, a separate analysis determines the conditions that are necessary and/or sufficient for the *negation *of an outcome. We found that the lack of high public social infrastructure is necessary for the lack of low loneliness (Online Appendix 2.4).^8^ In other words, whenever societies in Europe are not less lonely, they lack high public social infrastructure.

The above findings are elaborated on and contextualized within the following discussion section.

## Discussion

### Summary

Our goal was to identify how various forms of social infrastructure and the social composition of societies (in terms of their micro-level relationships) combine to impact loneliness outcomes among European societies. The most important findings—which are supported by robustness tests—involve the necessary condition “backbone” and three sufficient pathways toward a low degree of loneliness as well as a polarization between two key conditions.

#### Social Composition Matters, but Only in Combination with Social Infrastructure

We start to answer our research question by noting that a society’s social composition—the fact that individualist societies may have populations with “better” personal relationships in certain ways—is not a necessary condition for low loneliness in Europe. The social composition effect from aggregated strong personal relationships indeed provides an important component for achieving less loneliness in the two noncommercial pathways, but various types of social infrastructure are also crucial in combination with it. This supports part of Heu’s “culture-loneliness” framework (Heu et al. 2021) but highlights that social infrastructure is also required.

#### The Necessary Civil Society and Technological “Backbone” for a Society with a Low Degree of Loneliness

Overall, there are three pathways where the constructed and cultural features of social infrastructure interact with one another to achieve low loneliness in European countries. Most crucial from a policy perspective, because these conditions are more malleable, is that both *high internet access* and a *high degree of participation in associations* are necessary for societies in Europe to be “less lonely.” All three sufficient pathways share these conditions, so we label this the techno-civil social infrastructural “backbone” of loneliness alleviation.

The technological side of this shared techno-civil backbone involves high internet access, which appears to provide an essential degree of interconnectedness for the population. This is supported by recent work that has highlighted that technological development appears to strengthen social cohesion (Dragolov et al. 2016).

Equally important is the presence of a thriving civil society. Active volunteering appears to be an essential ingredient for less loneliness, presumably through the functions it provides directly to those who benefit from the voluntary activities as well as the social interaction that results for all parties involved in the voluntary activities, both between volunteers themselves (Lee 2021) as well as between volunteers and the people they support.

#### Three Pathways

In addition, three sufficient pathways toward a less lonely society are observed in Europe (Fig. 2). First, we have identified both *welfare support *and *cultural support *pathways, which share a high degree of strong aggregated micro-level social relationships in addition to the necessary conditions (internet and associations) and either public social infrastructure or cultural preferences for relational pluralism. The first path involves welfare state social protections and spending on social activities, and the second involves cultural preferences for universalism (relational pluralism). A third pathway involves the *commercial provision *of loneliness alleviation and requires the two necessary conditions, high commercial infrastructure, and the *negation* of public social infrastructure. Most countries that have a low degree of loneliness simultaneously follow the welfare support and cultural support pathways. However, welfare support and commercial provision are mutually exclusive—polarized—because the first requires high public infrastructure whereas the latter requires its negation (Fig. 2).

Although all three paths have similar parameters of fit, the welfare support and cultural support paths show the strongest logical relationship to a society with a low degree of loneliness (Online Appendix, Table 15).

The pathways to a low degree of loneliness for European societies are visualized in Fig. 2, where conditions are linked together within a logical network. Nodes in the network are the conditions involved in sufficient combination(s) (“pathways”) for low loneliness, and the links between nodes represent the copresence of these two conditions within a sufficient combination for the outcome. Each clique of mutually connected nodes, surrounded by the Venn-style boxes, signifies a sufficient combination for low loneliness. For example, since A, CI, I, and ~ PI are each connected with one another, they form a sufficient combination for LL. Locations of countries on the mapping can be read in the form of a Venn diagram, where overlaps between the three main policy boxes are meaningful. For example, Iceland and the UK are members of both the commercial provision and the cultural support pathways.

*Pathway 1: Welfare Support and Micro-Social Composition*

Welfare support loneliness mitigation involves a hybrid combination of the techno-civil backbone together with strong public social infrastructure and strong interpersonal relationships. Here, the public sector provides support for vulnerable individuals, presumably also the most socially isolated. It also creates opportunities for everyone for social engagement through its high level of funding for recreation, sports, and culture. In addition, these societies have strong interpersonal relationships that presumably mitigate loneliness by providing a strong degree of micro-level integration. As is the case for all paths, a high degree of internet access and high association participation combine with these two features to provide “backup” technological and social resources for those who lack an enhanced social network and/or cannot access public resources.

Norway and the Netherlands fit this welfare support pathway most consistently, and they are not simultaneously members of other paths. They had, respectively, the sixth and first lowest rates of loneliness in Europe in 2014. At the same time, both have very high rankings in public social infrastructure. Norway, for example, has the highest spending on social protection in Europe and the second highest spending on recreation, sports, and culture. These two societies rank, respectively, fifth and second for strongest personal relationships in Europe. They both also score exceedingly high in the techno-civil backbone, with the Netherlands leading all European societies in both internet access and volunteering rates. At the same time, these two societies do not belong to the second path, cultural support, because the extent to which they embrace relational pluralism is rather low.

This welfare support pathway is harmonious with the cultural support path toward low loneliness because six of its eight members are simultaneously members of both.

*Pathway 2: Cultural Support and Micro-Social Composition*

In 2014, Germany was eighth in Europe in terms of its per capita expenditures on social protection, but its spending on recreation, sports, and culture was only 15th out of 26. For this reason, Germany’s level in the multiplicative public social infrastructural index was too low for Germany to take advantage of the welfare support pathway. Instead, Germany has the fourth highest support for relational pluralism, a cultural preference for tolerance and openness toward diverse others. This, together with the fact that Germany follows only the cultural support pathway toward low loneliness, makes it an ideal representative.

The cultural preference for relational pluralism outlined above equates with the enhancement of individuals’ feelings of integration—and thus diminished loneliness—with others who are different from themselves. In addition, Germany has both of the necessary conditions for low loneliness. Its level of volunteering is third out of 26 in Europe. Its internet penetration was more modest in 2014, at 89%, but was still well above the middle of the pack. Germany also benefits from a social composition effect in that personal relationships are relatively strong, the eighth highest out of 26 countries in 2014. Both volunteering and a high cultural openness and tolerance for diverse others can translate into reduced stigma in terms of admitting feelings of loneliness and a greater sense of certainty that others have your best interests at heart. Because loneliness is a subjective phenomenon, the impact of such values should not be underestimated.

*Pathway 3: Commercial Provision*

The commercial provision pathway to reduced loneliness requires the *negation* of high public infrastructure in combination with high commercial infrastructure. Combined with the techno-civil backbone of high association participation and high internet accessibility, this pathway is in opposition to the welfare support pathway within the network of logical combinations sufficient for a low degree of loneliness (Fig. 2). By default, no society can occupy both paths at the same time, as they require opposite set memberships for high public infrastructure.

Ireland is the most typical case representing the commercial provision path, as it is the only society that occupies this pathway and no other. Ireland has the highest per capita expenditures on restaurants and cafes, which are presumed to provide a feeling of social integration through the social proximity and interaction with other customers, staff, and one’s own guests that arises through the sale of food and drink. At the same time, Ireland ranks only 14th out of 26 in terms of its level of public social infrastructure; its spending on social protection is rather low, and its expenditures per capita on recreation, sports, and culture are among the lowest in Europe, at 18th place out of 26. In terms of its techno-civil backbone, Ireland is just above the cutoff for internet access (at 82%) and does slightly better in terms of its association participation, as it is ninth in Europe. Ireland is the tenth “least lonely” society in Europe.

The explicit negation of public infrastructure as a necessary component of this pathway is intriguing. First, the withdrawal of state-provided social protection may allow for a profit-oriented, commercial social protection “market” to emerge. Second, this also suggests a lower degree of regulation of commerce so that it can pursue its sale of loneliness services without state interference. The sale of such social immersion by the consumer economy is presumed to provide people with a (purchased) feeling of integration, thus tentatively alleviating loneliness. Yet the combination of these factors with high association participation and high internet access suggests that this free-market provision of loneliness alleviation services is not sufficient on its own for reducing loneliness. Presumably, a high participation rate in volunteering and internet accessibility are necessary to “treat” loneliness among people who lack the financial resources to purchase interaction on the market.

The UK and Iceland are also members of this commercial provision pathway, but they simultaneously also belong to the cultural support pathway. Robustness testing reveals two further pieces of information about the commercial provision path. First, it is the least robust of those found. Only 27% of theoretically viable combinations of analytical decisions resulted in this exact pathway or its more specific subset appearing in a solution model. At the same time, a pairwise analysis of condition combinations confirms that *the core* of this pathway—a polarization between commercial and public social infrastructure—is robust (Online Appendix 3.5).

#### Polarization Between Public and Commercial Social Infrastructures

As noted above, there is a “configurational opposition,” or polarization, between commercial and public social infrastructures, as they are not found in combination within our analysis, their negation *is* found, and robustness checks confirm that it is much rarer for them to appear together within a solution model than to appear apart in various configurations. Analyzing this polarization is especially valuable because configurations are complex (involving many terms) and partially overlapping. This polarization opposes that trend. That the growth of commercial infrastructure dedicated to the sale of social integration services is achieved only when the public sector support is weakened might be analyzed by returning to theories that suggest the state as a counterforce necessary to counteract and dampen the more corrosive impacts of economic transformation upon social life (Polanyi 2001). In other words, while commercial infrastructure may dampen feelings of loneliness by selling the feeling of social interaction, the sustainability of this effect is in question because of the potentially corrosive nature of this economic activity.

Within the commercial provision path, the polarization between the state and commercial sectors is found in tandem with the techno-civil backbone. This is similar to how this backbone appears to serve as a safety net for people who do not benefit from public services, the goodwill of others, or strong social ties within the other two pathways. This claim is supported by our analysis for the negated outcome, *the lack of low loneliness*. In particular, Southern European societies had the high commercial infrastructure but negated public infrastructure combination that is key to the commercial provision pathway, but unlike Ireland, the UK, and Iceland, they had *high* levels of loneliness. This can be explained by the fact that they are missing the full techno-civil backbone; these societies each lack high internet access, high association participation, or both.

#### The Case of Slovenia

While Slovenia has quite low rates of loneliness (sharing the eighth lowest value with the UK), it remains unexplained. Like the UK and Iceland, it has universalist cultural preferences and high involvement in associations, but it lacks high enough internet coverage, strong personal relationships, and high commercial social infrastructure. As a result, it is not a member of any sufficient path.

In Slovenia, loneliness appears to be related to poor interpersonal relationships, which is also suggested by the fact that the lowest level of loneliness there seems to be among the married population (Lavrič et al. 2021). Yet we disregard an aggregation of this explanation, since the country has comparatively *low* marriage rates (Eurostat 2021e). Another explanation for Slovenia’s low degree of loneliness could lie in its topography: The nature-dense country (European Environment Agency 2017) is highly interconnected, with few remote rural regions and low-density urban settlements (DG Regional and Urban Policy 2015), potentially alleviating loneliness through easy access to nature and urban settings (Weinbrenner et al. 2021).

### Loneliness Policy

Some societies have redundant social resources for dealing with loneliness because they are located on multiple pathways that may alleviate loneliness (Fig. 2). Denmark, Sweden, Finland, Belgium, Austria, and Switzerland are members of pathways one and two, which may provide them with more options for managing loneliness. Such *condition redundancy* suggests a more robust capacity for dealing with loneliness.

An intentional state-level “loneliness policy” would do well to maximize its redundancy in this sufficiency network by focusing on the techno-civil backbone and avoiding the polarization between public and commercial infrastructure. Given the comparative strength (in robustness as well as case membership) of the welfare support and cultural support pathways, the opposition between commercial and public social infrastructure, evidence that welfare spending seems to further enhance the necessary condition of volunteering (Ackermann et al., this issue), information about the negated outcome showing the crucial role of public infrastructure, and robustness checks supporting the above, the policy implication is clear. An informed loneliness management policy should, besides the techno-civil backbone, especially foster public social infrastructure through protecting vulnerable populations and maximizing state spending on social activities (see Nyqvist et al. 2019).

### Limitations and Future Work

Theoretically, it is possible that loneliness is *less stigmatized* in individualist societies, and this may play some role in people feeling less lonely. However, the opposite may also be the case because individualist societies are defined by their high valuation of independence. Loneliness—the feeling that social ties are inadequate—in such a setting might be seen as the admission to oneself and others that one has “failed” in achieving an ideal independence from others. As a result, it is possible that individualist societies only appear to be less lonely because they entail *more stigma,* and lonely people are less likely to report it. While we do not discount this possibility, if it were true, it would likely not represent the whole picture. To interpret our results through such a frame would amount to an attempt to interpret our three paths in terms of how they each lead, in specific ways, to an enhanced loneliness-reporting stigma. Theoretically, this would be a tall order.

Because our sample represents a wide variety within Europe, applying our frame to other European societies should be considered. Beyond Europe, the nature of QCA implies that it is possible that different pathways than the ones we have identified will be found as the cases involved (and time frames covered) shift.

We are also aware of the limitations of our data in terms of country scope, time frame, and the specific loneliness measures used. It would be valuable to check findings against a broader sample, but the World Values Survey lacks questions on loneliness, and the International Social Survey Program, which asked about loneliness in 2017, has too limited a country sample (Taniguchi and Kaufman 2021). Our data are limited to the 2014–2015 period. It would, of course, be valuable to see how loneliness itself as well as its contributing conditions toward its resolution have shifted over time. If the ESS includes loneliness questions again in upcoming waves, this would become possible.

Inquiring about a subjective phenomenon such as loneliness through a single indicator has limitations. More nuanced ways of measuring loneliness in cross-national surveys would be the de Jong Gierveld six-item or eleven-item scales (de Jong Gierveld and van Tilburg 2006), which are able to measure overall loneliness in addition to the subcategories of emotional and social loneliness.

Future QCA as well as traditional quantitative studies might consider local or regional-level comparisons in order to study social infrastructure findings at a lower level of aggregation. In addition, more qualitative work focused on loneliness resolution and management in daily life would also be a welcome contribution to help concretize the mechanisms by which the condition combinations we have identified here actually unfold.

The current evidence challenges concerns about an overarching “loneliness pandemic” in modern societies. Yet few efforts have gone into understanding why and how the most individualist societies are less lonely. Our findings suggest that they benefit from diverse social resources for managing loneliness through a vibrant civil society, access to the internet, and public protection of the vulnerable as well as funding for social activities. We have found reasons to doubt the effectiveness of commercially sourced loneliness alleviation, as it does not appear to be robust, and at the same time it seems to come at the expense of welfare support, which our research suggests is the surest route toward a less lonely society in Europe.

## Footnotes

1 The cross-level interaction effects suggested by the framework, by which the alignment of personal and cultural norms may reduce loneliness, are not substantiated by evidence from the 2014 wave of the European Social Survey. Also, the aggregation of social isolation does not fully explain individualism’s impact on loneliness (Swader 2019, pp. 1327, 1331).

2 We also differ from de Jong Gierveld and Tesch-Römer’s (2012) macro-level depiction in that, in line with our definition of social infrastructure, we consider cultural preferences to be a form of social infrastructure.

3 An INUS condition is an insufficient but necessary part of a combination of conditions that is itself unnecessary but sufficient for the outcome (Schneider and Wagemann 2012, pp. 78-79). In other words, such conditions are not expected to give rise to the outcome *by themselves*, but they may be part of a *group* of conditions that is sufficient for the outcome. Being “sufficient for an outcome” means that when this combination is present, the outcome is also present; however, other sufficient paths to the outcome may exist. We also highlight that, within QCA, which emphasizes contingency, it would be extraordinarily rare for a condition *on its own* to be sufficient for an outcome.

4 Note that QCA does not use “variables.” In contrast to a variable, a condition refers to a membership in a defined set. For example, “height” is a variable, but “very tall” or “average height” would be related conditions.

5 When using negations, we will use the tilde in front of the negated condition/outcome: ~ X.

6 Our analysis was conducted in *RStudio* using the “QCA” (Dușa 2019) and “SetTheoretic” (Oana and Schneider 2018) packages in addition to our own *R* script for the configurational robustness checks.

7 The intermediate sufficiency solution is *CI*I*A*~* *PI* *+* *I*A*CP*P* *+* *I*A*P*PI* → *LL*.

8 Necessity formula for the negated outcome: *~* *PI ← ~* *LL.*
